# Meliotropic wellbeing mindset: a catalyst for sustaining long-term wellbeing

**DOI:** 10.3389/fpsyg.2025.1637864

**Published:** 2025-11-20

**Authors:** Jolanta Burke, Andrea Giraldez-Hayes, Padraic James Dunne

**Affiliations:** 1 Royal College of Surgeons in Ireland, Dublin, Ireland; 2 University of Cambridge, Cambridge, United Kingdom

**Keywords:** meliotropism, meliotropic wellbeing mindset, wellbeing, positive psychology, sustainable wellbeing, alumni of positive psychology, long-term wellbeing, positive psychology intervention (PPI)

## Abstract

Positive psychology has contributed significantly to wellbeing, primarily through Positive Psychology Interventions (PPIs). However, most research in PPIs has focused on their impact on positive outcomes rather than the conundrum of real-life application. This qualitative study explored how 22 alumni of positive psychology programmes integrated their wellbeing knowledge into daily life to sustain their wellbeing over time. Participants reported a shift from using structured PPIs for enhancing their wellbeing to a new mindset that supported their long-term wellbeing. Specifically, five themes have emerged from the data, which represented manifestations of their changed mindset: (1) intentional living, (2) wellbeing hygiene, (3) self-acceptance, (4) embodiment, and (5) environmental awareness. These themes describe “Meliotropic Wellbeing Mindset,” which is an intrinsically motivated, wholly approach to living a good life that integrates wellbeing into everyday practice through planned or spontaneous, context-sensitive, and identity-aligned practices. These findings challenge the field’s focus on PPIs and suggest that sustainable wellbeing is more about developing mindsets that orient individuals toward continual improvement than practising PPIs. Deriving from the Latin “melior” (better) and Greek “tropism” (movement toward), Meliotropism describes a tendency to actively align thoughts, behavior, attitudes, and life choices with what makes life worth living, even amid adversity. This research highlights the importance of fostering Meliotropic Wellbeing Mindset in real-world contexts, where wellbeing is an ongoing, self-directed, and contextually responsive process.

## Introduction

Positive psychology has grown exponentially over approximately three decades (Hart and Sasso, 2011; Kim et al., 2018; Rusk and Waters, 2013). One of its most notable contributions is the application of wellbeing toward addressing the United Nations Goal 3 (United Nations, 2024). This application is mainly focused on Positive Psychology Interventions (PPIs), often referred to as Wellbeing Activities (WA) or Positive Activities (PA) (Burke et al., 2023; Parks and Schueller, 2014). They are a foundation of many wellbeing programmes (e.g., Hendriks et al., 2020; Tejada-Gallardo et al., 2020), including multidisciplinary approaches to enhancing wellbeing (e.g., Burke and Dunne, 2024; Przybylko et al., 2021). The leading model for implementing them encourages the Person-Activity fit whereby the person’s characteristics, such as motivation, effort, personality or social support, need to align with activity features, such as dosage, variety or time perspective (Lyubomirsky and Layous, 2013). However, recently emerging research testing the model questions some of its initial assumptions, such as variety, too much of which leads to disengagement (Okabe-Miyamoto et al., 2021). Thus, significantly more research is required to understand the mechanisms for PPI’s effective application.

In the meantime, strong evidence exists of the positive impact of wellbeing interventions, such as an increase in life satisfaction, wellbeing, strengths, and quality of life, as well as a decrease in anxiety, stress and depression at small to moderate levels (Carr et al., 2020, 2023; Donaldson et al., 2011; Hendriks et al., 2020). Furthermore, PPIs show the evidence of enhancing wellbeing and reducing distress of clinical patients with such conditions as cardiovascular disease, coronary artery disease, chronic pain, or schizophrenia (Blasco-Belled et al., 2023; Pina et al., 2021; Tönis et al., 2023), although the research has been primarily conducted in a lab environment. Research translation into real life continues to be challenging (Ryff, 2022). Subsequently, models have been developed to ease the real-life application. They include systems-informed approaches that consider the complexities associated with practising interventions, wellbeing literacy as a potential contributor to long-term wellbeing, or a multidimensional and multi-level extended evolutionary approach to practice (Ciarrochi et al., 2022; Kern et al., 2019; Oades et al., 2020). The gap continues to exist about the applications of positive psychology in real life, which the current study aimed to address.

While most research on the application of positive psychology focuses on exploring the use of PPIs and their impact, one of the challenges practitioners face is the sustainability of applying positive psychology practices (Wong and Roy, 2022). Practising PPIs as once-off interventions or part of a programme is helpful, but their repetition can be wearisome and may result in a decline in wellbeing (Lyubomirsky et al., 2005). Thus, other practices have been developed to avoid this. For example, creating programmes that intertwine health (e.g., nutrition, movement) with PPIs (Przybylko et al., 2021), strategies maximising positive experiences by dialling up the positive emotional responses to them (Quoidbach et al., 2015), or tapping into the concept of wellbeing literacy that plays a role in the effectiveness of wellbeing outcomes (Oades et al., 2020). However, further research is required to understand how PPIs can be used in the application of positive psychology research and how they can be applied beyond the use of PPIs. This ongoing need for research presents an opportunity to engage in the ongoing development of positive psychology.

One way to explore this topic is by sampling alumni of positive psychology programmes, who are trained experts and practitioners of wellbeing interventions and who possess wellbeing literacy (Oades et al., 2020), enabling them to make more informed and intentional wellbeing choices. Currently, 61 institutions around the world offer positive psychology programmes (Wang et al., 2023). While curricula vary, the core content remains consistent. Previous research has shown that positive psychology training enhances university students’ wellbeing by enhancing their self-awareness, emotional intelligence, and providing a deeper understanding of how to apply wellbeing principles in their lives (Hobbs et al., 2024; Hood et al., 2021). Furthermore, a recent systematic review demonstrated that studying positive psychology can improve students’ wellbeing by increasing their life satisfaction and happiness (Hobbs et al., 2022). These results highlight the great value of positive psychology for supporting wellbeing.

These findings also demonstrate that both PPIs and the academic study of positive psychology equip individuals with practical strategies for enhancing wellbeing. However, none of these studies have explicitly focused on students who have completed a Master’s in Applied Positive Psychology (MAPP), and who, through their studies, have developed advanced wellbeing literacy and experience in applying PPIs over time. This positions them as a distinctive group for exploring how positive psychology knowledge is applied beyond the structured PPIs typically recommended in practice.

Accordingly, the current study explored how these positive psychology experts and practitioners (MAPP alumni) integrate their knowledge of positive psychology into daily life. Given the central role of interventions in positive psychology, the study also examined their use of PPIs. The guiding research question was: *How do alumni use their knowledge of positive psychology in everyday life?*

## Methods

This qualitative research design comprised online, in-depth, semi-structured interviews.

### Participants

A total of 22 participants took part in online interviews. They had all completed an online survey about “Flourishing Alumni” and had consented to be contacted for the second stage of the research project, which involved these interviews. The online survey comprised 170 participants, aged *M* = 47.2 (SD = 9.5), located mainly in the UK (56%), followed by the EU (16%), USA (4%), Australia (2%) and the remainder were located elsewhere. Participants were mainly female (92%). Their positive psychology qualifications were Master’s in positive psychology (82%), followed by a PhD (5%) and all other qualifications, such as cert, or diploma in positive psychology. The current study participants were all female, aged between 29 to 66; the average age was 48. Only two participants had less than a year’s experience in practising positive psychology. Thirteen participants had 1–4 years, five of them had 5–10 years, and two had over 10 years of experience. Regarding education, 19 participants had a Master’s degree in Positive Psychology, two had a Diploma or Certificate, and one held a PhD. Eleven participants were based in the UK, four in Ireland, and seven in the European continent. There were no participants from outside of Europe. Although the final interview sample consisted exclusively of female and EU participants, considerable effort was made to ensure gender and geographical diversity. Several male participants, who were drawn from the initial quantitative “Flourishing Alumni” survey, expressed interest in the follow-up interviews. Following the ethical guidelines, we contacted each interested participant once and followed up with two reminders. Those who did not respond within this timeframe were not contacted further to respect their autonomy and avoid undue pressure. Despite this, none of the male participants completed the interview phase of the “Flourishing Alumni” study, leading to the sample comprising female participants only. This gender-homogeneous outcome was not surprising given that in our quantitative sample, 92% of respondents identified as female. While gender imbalance is a limitation of the current study and should be addressed in future research, it reflects the demographic reality of the field and does not negate the value of the insights gained from this study.

Regarding geographical homogeneity, with all participants based in the EU, this outcome was primarily due to practical and logistical factors, including time zone compatibility and responsiveness to interview scheduling. Following our participant contact protocol, we reached out to interested individuals up to three times. Participants outside the EU have either not responded within the specified timeframe or have not been able to schedule a suitable time for an interview. This geographical homogeneity limits the generalisability of the findings to non-European contexts. However, it is important to note that qualitative research is not intended to produce statistically generalisable findings. Instead, its purpose is to generate rich, in-depth insights into participants’ lived experiences, meanings, and perspectives, which is what the current study addressed.

### Procedure

Ethical approval (ETH2122-0255) was obtained from the Ethics Committee of the School of Psychology, University of East London. Only participants who have consented to research were included in this study. Before an interview, participants completed an online survey and agreed to participate in a follow-up interview. A research assistant contacted all participants who consented to participate in the following research stage and arranged interviews with the leading author. Interviews were conducted and recorded using Microsoft Teams.

A qualitative methodology was chosen to capture the nuanced, subjective experiences of participants in applying positive psychology in real life. This approach is particularly well-suited for exploring emergent constructs such as mindset transformation, where depth, context, and personal meaning are central (Brown and Clarke, 2022). The interview schedule comprised one main question: “How do you use positive psychology in your daily life?.” Prompts included enquiring about participant use of positive psychology at various stages of their lives, how they got interested in it in the first place, what they found most valuable about it and what change, if any, they have noticed about themselves since practising positive psychology.

Data was transcribed by an RA via Otter, anonymised, ensuring pseudonyms have been used during the analysis stage. Recordings were deleted as soon as they were transcribed, and data was uploaded onto MAXQDA (version 2022) for analysis. Preceding and following each interview, the researcher engaged in a reflective practice, which included participant observations, impressions, researcher’s preconceptions, and emotional responses evoked during the interview. Reflective notes were referred to during the research analysis stage. The interview data was entered into the MAXQDA software. Thematic analysis was conducted inductively following Braun and Clarke’s six-phase approach (2019). Initial coding was performed line-by-line by the first author using MAXQDA. The first and second co-authors independently reviewed a subset of transcripts to discuss interpretative discrepancies. Themes were refined through iterative discussion among the research team until consensus was reached. Data saturation was considered complete when no new themes emerged in the final five interviews. Triangulation was supported by reflexive journals maintained by the interviewer and the first author, alongside a review of post-interview notes.

## Results

**Figure 1:**
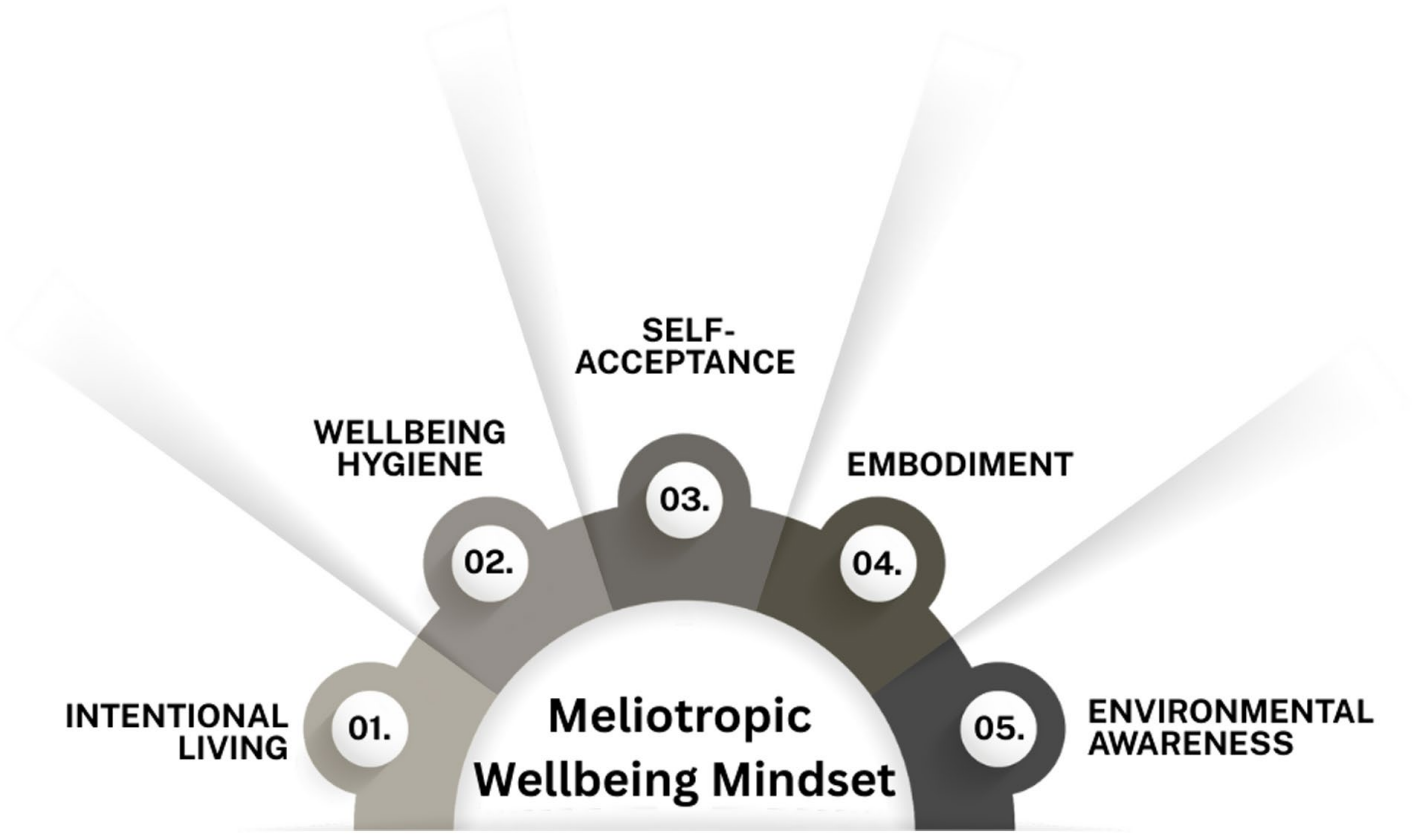
Five themes representing manifestations of Meliotropic Wellbeing Mindset.

Five themes emerged from data: (1) intentional living, (2) wellbeing hygiene, (3) self-acceptance, (4) embodiment, and (5) environmental awareness (see Figure 1).

### Intentional living

Participants described a mindset shift that led them to integrate positive psychology into their daily lives, not through structured interventions, but rather via intentional orientation towards wellbeing.

“So it’s kind of like the new mindset that you have” (17 Julia, Pos. 48)

While many alumni initially engaged with PPIs during their education, most of them no longer practised them. Instead, they preferred engaging in more spontaneous, and meaningful actions aligned with their personal values. This shift reflected a preference for flexibility in how they engaged with daily tasks and authenticity in their approach, instead of practising prescriptive wellbeing interventions.

However, rather than rejecting PPIs entirely, participants acknowledged their value, especially as tools recommended to clients who were new to positive psychology. Nonetheless, they preferred to act on their positive psychology knowledge that was transforming their actions organically. This often involved informal, situation-specific application of wellbeing strategies, such as reflection during transitions or emotion regulation during challenges. The intentional mindset was not defined by rigid daily practices but by value-driven awareness of opportunities presenting themselves in a moment that may have supported their wellbeing.

“I don't really use PPIs, it's more for me a way of thinking. So, I would say that I recommend them to others. And I can kind of talk about my own experience of having used them.” (17 Julia, Pos. 16)

Although they did not actively pursue wellbeing or evaluate their wellbeing levels via assessments, participants described having a heightened attentiveness to how their actions aligned with their goals and what their life purpose was. Intentional living, therefore, was not about constant practice of PPIs or self-monitoring but about allowing wellbeing literacy to inform their daily decision-making in a flexible, self-aware way.

### Wellbeing hygiene

Participants reported introducing small actions into their lives that contributed to their sustained wellbeing. These actions, such as preparing meals with care, engaging in hobbies, practising creativity or mindfulness, were not identified as structured PPIs, but rather as “hygiene” practices that reinforced their wellbeing orientation.

“Having the time to cook dinner properly (…) could be kind of humdrum, boring run-of-the-mill things, but actually can be done in a way that's really lovely and can contribute to, so many different aspects of wellbeing.” (5 Helen, Pos. 41)

Participants also highlighted the social scaffolding, being an important part of their wellbeing hygiene. This involved intentionally staying connected with others who shared their wellbeing values. Maintaining friendships with fellow MAPP graduates or joining communities of practice that help them remain grounded and continue their personal development.

“The biggest thing is that (…) I've made some incredible friends. And that is really lovely, because you're then in a group, and you can talk about, hope theory or resilience or all the different kinds of the language we use.” (16 Margaret, Pos. 52)

For many participants, wellbeing hygiene also included making alternative career choices, adjusting their lifestyle and daily practices that allowed them to integrate wellbeing within their personal and professional lives. These daily choices, which ranged from introducing knitting to engaging with their clients differently, reinforced their belief of the importance of cultivating wellbeing daily.

### Self-acceptance

Participants reported their growing comfort with their whole selves, which included accepting their vulnerabilities and coming to terms with struggles, be it related to health, relationships, or life circumstances. They no longer strived for constant positivity. Instead, participants learned to accept emotional lows, embracing their “shadows,” and accepting their imperfection as being part of the human experience. Positive psychology became a framework through which they could process difficulty, rather than bypass it.

“It (positive psychology) gave me a knowledge of who I am and an ownership of my thoughts and feelings and behavior.” (21 Martha, Pos. 50-52)

Participants described this self-acceptance as liberating. It allowed them to make decisions in line with their authentic selves, and to experience wellbeing as something generated internally rather than it being externally imposed. They prioritised practices that helped them feel balanced, even when they faced adversity.

“Sometimes you cope pretty and sometimes you cope ugly and coping ugly might be crying and eating a bag of cookies.” (1 Laura, Pos. 94)

Importantly, their sense of agency has grown alongside their self-acceptance. Rather than reacting impulsively to distress, participants became more skilled in self-regulating their thoughts and emotions. They also became more discerning about what supported or hindered their wellbeing. For them, authenticity included owning discomfort, asking others for space, and making their own space for rest and recovery.

### Embodiment

Participants consistently reported an increased awareness of the body and mind connection. They described this integration as key to supporting their wellbeing. Their daily practices extended beyond cognitive or emotional strategies they learnt during their positive psychology courses, and included physical and sensory awareness. Movement, nutrition, sleep, and engaging with nature were not just part of their health-focused behavior. Instead, they were deeply connected to their experience of psychological flourishing.

“It is positive psychology to move your body and it is positive psychology, I think, to sleep and to eat well.” (1 Laura, Pos. 105)

Rather than pursuing high-intensity exercise or introducing dramatic lifestyle changes, participants tended to describe routines grounded in balance, such as alternating between running, yoga, and walking. They spoke of embodiment as a practice of syncing the body and mind through small rituals, like mindful coffee or spending time in nature.

“From the start of the morning I am getting my body and my mind kind of to the same page.” (2 Karen, Pos. 6)

Embodiment has served participants as both a wellbeing practice and a metaphor for integration. For them, the internalisation of positive psychology became physical, physiological and sensory, not just affective, psychological or theoretical. This physical grounding gave participants a stable base from which they were able to navigate daily challenges and sustain their wellbeing mindset.

### Environmental awareness

Finally, participants have expressed their increased sensitivity to the environment, whether physical or social, and the influence it has had on their wellbeing. They made deliberate choices about where they worked, whom they spent time with, and how they managed their physical and emotional boundaries. For many, leaving toxic workplaces or limiting their time with negative individuals was perceived not as avoidance, but as a proactive act of self-preservation.

“I can say no to all the things that I'm not interested in…I actively spend time with people who want to learn and do things differently.” (11 Claudia, Pos. 42)

They also extended their environmental discernment outwardly. Participants saw their behavior as being potentially influential in impacting the wellbeing of others. By modelling positivity, resilience, or self-compassion, they hoped to inspire similar orientations among people in their families, workplaces, and within their communities. To them, this environmental awareness has become a form of wellbeing ecology, whereby they shaped their environment to align with their values. Thus, participants viewed their relationships and physical environment not only as contexts but as co-constructors of their wellbeing.

## Discussion

Our research aimed to explore how alumni who are experts and practitioners in positive psychology apply knowledge to enhance their own wellbeing. The findings revealed a discrepancy between the recommended practices in the positive psychology literature and the actual daily use of positive psychology by these experts and practitioners. While existing research strongly advocates for PPIs as key tools for improving wellbeing, and promotes models such as Person-Activity fit to enhance their effectiveness (Lyubomirsky and Layous, 2013; Parks and Schueller, 2014; Pawelski, 2020), our findings suggest that these approaches may not be sufficient for sustaining long-term wellbeing. Participants reported initially engaging with PPIs to understand how they work, but confirmed that these practices rarely remained part of their daily routines. Instead, they developed and adopted a range of value-integrating practices that better supported their orientation to living a good life.

The most significant and novel finding of this research is the shift in participants’ mindsets that developed over time through their engagement with positive psychology. This new orientation towards wellbeing reflected an intrinsic drive to pursue what constitutes a good life, even in the face of difficulty. Rather than focusing on the pursuit of happiness or the avoidance of pain, this wellbeing-orientation mindset allowed participants to embrace the full spectrum of human experience while consistently leaning toward growth and flourishing. Similar patterns have been observed in the context of Positive Health, which they describe using the term *Meliotropism* (Burke et al., forthcoming; Dunne et al., 2025). Derived from the Latin “melior,” meaning “better” or “mostly well,” and Greek “tropism,” referring to an organism’s movement toward a stimulus, Meliotropism describes the tendency to orient oneself toward wellbeing and flourishing (Dunne et al., 2025). The current research extends these insights by emphasising the transformative role of what we refer to as a *Meliotropic Wellbeing Mindset* in everyday life. This mindset is shaped by intraspective factors (internal thoughts, beliefs, emotions, and mindset), interspective factors (relationships and social connections), and environmental influences (physical, cultural, and systemic contexts). This mindset is also the main driver of sustainable wellbeing in the participants who were experts and practitioners of positive psychology.

In our research, one of the features of the Meliotropic Wellbeing Mindset was participants’ belief in an increased sense of perceived control over their wellbeing, whilst at the same time having an awareness of the limitations of that control. This balance may help explain their reported increase in confidence, which aligns with past research indicating links between perceived control and confidence (Johnson and Kilmann, 1975). For some participants, this new mindset was grounded in an increased sense of authenticity and an unapologetic approach to making life choices that supported their wellbeing orientation. Interestingly, none of the participants mentioned the pursuit of happiness as a goal, which research has shown to be associated with adverse wellbeing outcomes (e.g., Gruber et al., 2011). Instead, they displayed a high level of self-awareness regarding both their own needs and those of others, making thoughtful decisions that enhanced wellbeing by actively seeking everyday opportunities to live a meaningful and fulfilling life. This shift in attitude is the foundation of the Meliotropic Wellbeing Mindset, which warrants further investigation in real-world contexts.

Another essential aspect of the Meliotropic Wellbeing Mindset that emerged from this research was participants’ full acceptance of self, both the positive aspects of their personality, life and experiences and their negative aspects. They did not deny their “shadow” (Casement, 2003), nor did they focus solely on their positive experiences. While they valued good mental and physical health, they were fully aware of their negative experiences and emotions and explored ways in which they could balance their lives, instead of focusing on increasing happiness. This contrasts with the criticisms of positive psychology as a happyology that focuses on the positive sides of human experience only (van Zyl et al., 2024). Other researchers have highlighted similar concerns about the field’s early overemphasis on positivity (Ryff, 2022; Wong, 2011), cultural universality (Kristjánsson, 2012), and failure to address adversity and suffering in meaningful ways (Lomas and Ivtzan, 2016). The reason could be either the misunderstanding of positive psychology research by those who criticise it or the nature of the European positive psychology courses that the 22 participants of the qualitative research attended, which is primarily based on the second wave positive psychology that acknowledges the need to combine all life experiences, including positive and negative (Lomas and Ivtzan, 2016). Further research is required to explore the Meliotropic Wellbeing Mindset intricacies that drive sustainable wellbeing change with the general public. Perhaps refocusing the field of positive psychology away from lab-led research of PPIs with short-term effects to Meliotropic Wellbeing Mindset that drives sustainable change could reduce the burden of mental health issues highlighted by the World Health Organisation (2022).

The link between the body and mind was another unique finding that presented Meliotropic Wellbeing Mindset as all-encompassing. Positive psychology focuses primarily on the mind, yet participants were very much aware of their bodies and improved their body care after their positive psychology education. They began to see the body and mind as one, indicative of monistic thinking about the body–mind connection, as opposed to Descartes’ duality of body and mind (Riekki et al., 2013). Research indicates that monism supports better health outcomes (Burgmer and Forstmann, 2018; Forstmann et al., 2012). However, little is known of the outcomes of monism on wellbeing. Thus, further research should explore this link and examine why the impact of positive psychological training resulted in monistic thinking among the alumni of positive psychology.

It is important to note that the Meliotropic Wellbeing Mindset, as identified in this study, is not a mere adaptation to knowledge acquisition. While knowledge acquisition is a common feature of learning (Donovan et al., 2000; Illeris, 2009), the changes reported by participants in this study reflect a deeper, transformative shift consistent with theories of experiential and transformative learning (Kolb et al., 2001; Taylor, 2007). This suggests that the Meliotropic Wellbeing Mindset is not just a reaction to exposure, but a durable, life-altering shift in participants’ orientation toward life and wellbeing.

Knowledge acquisition involves gaining knowledge and skills (Schunk, 2012). However, the participants of the current study also developed their enduring disposition toward wellbeing, a new lens through which they began to interpret, navigate, and design their lives. It is, therefore, less about their wellbeing literacy, inspired by knowledge acquisition and more about the choices they make in how they live their lives (Ryan and Deci, 2000; Taylor, 2007). Acquiring knowledge does not always result in behavioral change due to motivational or contextual challenges (Sheeran et al., 2016). For example, people may know that exercise is good for them, but they do not act on it. However, in the current study, alumni did not merely understand wellbeing; instead, they have interwoven it into their daily routines, decision-making, and spontaneous acts, supporting their wellbeing. This transformational change reflected their embodiment and full integration of wellbeing and identity change as alumni in positive psychology, not just their intellectual or emotional growth.

Unlike passive or extrinsically motivated knowledge acquisition, the Meliotropic Wellbeing Mindset is intrinsically motivated. Participants oriented themselves toward wellbeing because it aligned with their aspiration of who they wanted to become. Importantly, many participants adopted this mindset *after* completing their positive psychology programmes. In contrast, knowledge acquisition typically peaks during formal education and may decline without reinforcement (Schunk, 2012). The Meliotropic Wellbeing Mindset is self-reinforcing and sustainable, ensuring its long-term benefits. Thus, this new concept is an apt description of changes that emerged in the alumni, which go beyond knowledge acquisition or wellbeing literacy.

Furthermore, Meliotropic Wellbeing Mindset demonstrated a sustained, intentional orientation toward wellbeing that had become deeply integrated into participants’ ways of thinking, relating, and living. This mindset was not limited to the adoption of techniques or isolated behavior changes. Instead, it reflected an internalised framework for navigating life, consistent with the principles of second-wave positive psychology (Lomas and Ivtzan, 2016; Wong, 2011). Such reflective transformation also aligns with Dweck’s (2012) definition of mindset as a stable, self-reinforcing orientation that influences perception, motivation, and behavior across contexts.

Moreover, the Meliotropic Wellbeing Mindset provides a comprehensive, integrative lens that unites three domains of wellbeing engagement: intraspective (e.g., self-awareness or self-regulation), interspective (e.g., value-aligned social networks), and environmental (e.g., context-sensitive decision-making). While each of these aspects has been explored independently in existing wellbeing literature (e.g., Ciarrochi et al., 2022; Ryan and Deci, 2000; Ryff, 2022), the present study highlighted how these elements converge into a lived, real-life, self-sustaining orientation toward flourishing that persists without reliance on structured interventions, regardless if they are psychological or educational.

### Implications for research and practice

These study findings have significant implications for future research and practice. Firstly, researchers should consider exploring more topics related to the process of incorporating positive psychology into daily life rather than focusing on evaluating various interventions and encouraging their use based on research outcomes. It might be time to rethink how positive psychology is taught and practised and potentially move away from the current approaches of designing programmes comprising hours of PPI practice. For educators in the field, this study highlights the need to shift their focus from teaching about interventions to focusing on other pathways for integrating positive psychology research into students’ everyday lives, such as a Meliotropic Wellbeing Mindset. This approach may result in a more long-lasting impact of positive psychology research on individuals’ and community’s wellbeing. However, more research is required to confirm this.

Instead of relentless engagement in a range of wellbeing programmes, interventions that help people incorporate Meliotropic Wellbeing Mindset into their daily lives could become a stepping stone towards practising more sustainable wellbeing. This nuanced approach to practising positive psychology may be significantly more effective than relying on a range of PPIs. However, significantly more research is needed to explore the complexities of the Meliotropic Wellbeing Mindset, particularly its role as a moderating factor in improving long-term wellbeing.

### Limitations

This study has three main limitations. Firstly, the data was collected from positive psychology alumni who were already well-versed in wellbeing literacy due to their education. Future research should replicate this study by exploring the mindsets of the general public who lead fulfilling lives based on positive psychology principles. This will allow them to determine whether similar thinking patterns are present in the general population. Secondly, this study findings were drawn from 22 participants. While this is a substantial and adequate number for qualitative research, a much larger sample size is required to generalise these findings. Lastly, all participants in this research were from Europe, meaning the impact of positive psychology education assessed in this study may be specific to European positive psychology courses. Further research should include positive psychology alumni from other continents to gain a more global perspective on the use of positive psychology by wellbeing experts and practitioners.

## Conclusion

Despite its limitations, the current study makes a unique contribution to the existing body of research by challenging both researchers and practitioners to reconsider how positive psychology knowledge is introduced and developed over time in real-life. Importantly, it introduces the novel concept of a Meliotropic Wellbeing Mindset, offering a foundation for deeper exploration of positive psychology and its application in ways that promote sustainable wellbeing. The study also highlights the importance of integrating the body into positive psychology education, highlighting its critical role in supporting alumni’s daily wellbeing practices.

## Data Availability

The raw data supporting the conclusions of this article will be made available by the authors, without undue reservation.
